# Multi-modal ultra-high resolution structural 7-Tesla MRI data repository

**DOI:** 10.1038/sdata.2014.50

**Published:** 2014-12-09

**Authors:** Birte U Forstmann, Max C Keuken, Andreas Schafer, Pierre-Louis Bazin, Anneke Alkemade, Robert Turner

**Affiliations:** 1 Amsterdam Center for Brain & Cognition, University of Amsterdam, 1018 WS Amsterdam, Netherlands; 2 Max Planck Institute for Human Cognitive and Brain Sciences, 04103 Leipzig, Germany

## Abstract

Structural brain data is key for the understanding of brain function and networks, i.e., connectomics. Here we present data sets available from the ‘atlasing of the basal ganglia (ATAG)’ project, which provides ultra-high resolution 7 Tesla (T) magnetic resonance imaging (MRI) scans from young, middle-aged, and elderly participants. The ATAG data set includes whole-brain and reduced field-of-view MP2RAGE and T2*-weighted scans of the subcortex and brainstem with ultra-high resolution at a sub-millimeter scale. The data can be used to develop new algorithms that help building high-resolution atlases both relevant for the basic and clinical neurosciences. Importantly, the present data repository may also be used to inform the exact positioning of electrodes used for deep-brain-stimulation in patients with Parkinson’s disease and neuropsychiatric diseases.

## Background & Summary

Large collaborative projects between scientific groups spread around the world are aimed to increase our understanding of the human brain. Large human connectome studies^1–3^ are in place working to clarify the connectivity within the human brain using a multi-modal approach ranging from structural brain imaging to genetics (http://www.humanconnectomeproject.org). However, to fully understand the connectivity of the brain, we need a higher level of anatomical detail than currently available. The lack of knowledge about small brain structures, especially subcortical structures, is reflected by their absence from brain atlases currently available for MRI research^4,5^. A comparison of subcortical grey matter structures depicted in standard MRI-atlases with the structures defined in the Federative Community on Anatomical Terminology^6^ yielded an overlap of only seven percent. One important explanation for this discrepancy is the absence of ultra-high resolution MRI data allowing the direct visualization of small nuclei in the subcortex. A second important reason is the lack of automated analytical protocols available for MRI-data segmentation, with the resulting necessity of laborious studies performed by trained anatomists for the identification of subcortical brain areas. Thirdly, besides the lack of anatomical knowledge, there is no information about age-related changes in, e.g., volume or location of subcortical structures.

Recent exciting advancements in the field of ultra-high resolution magnetic resonance imaging at 7 Tesla (or higher) allow *in vivo* neuroimaging of the human brain with unprecedented anatomical detail^7–11^. Here we share information of a multi-modal data set of three different groups of young, middle-aged, and elderly participants who were scanned with a 7 T MRI scanner. The data sets contain three different age groups and can be used to investigate anatomical changes due to healthy aging. The data sets have already been used to create probabilistic atlas maps including the striatum, globus pallidus interna and externa, the substantia nigra, the subthalamic, and the red nucleus. All probabilistic atlas maps are available online (https://www.nitrc.org/projects/atag/ and http://fsl.fmrib.ox.ac.uk/fsl/fslwiki/Atlases). In addition to the manual segmentations, the data can be used to develop new algorithms that help building high-resolution subcortical brain atlases that can be directly applied in both the basic and clinical neurosciences. Finally, the data can be used to guide the exact positioning of electrodes relevant for deep-brain-stimulation often used in patients with Parkinson’s disease and neuropsychiatric diseases^12–14^.

## Methods

### Participants

For the acquisition of the structural brain scans, 30 young participants (14 females) with mean age 23.8 (s.d. 2.3), 14 middle-aged (7 females) with mean age 52.5 (s.d. 6.6), and 10 elderly (3 females) with mean age 69.6 (s.d. 4.6) were included (Table 1). All participants had normal or corrected-to-normal vision, and none of them suffered from neurological, psychiatric, or somatic diseases. All subjects were right-handed, as confirmed by the Edinburgh Inventory^15^. The study was approved by the local ethics committee at the University of Leipzig, Germany. All participants gave their written informed consent prior to scanning and received a monetary compensation.

### Scan parameters

The structural data were acquired using a 7 T Siemens Magnetom MRI scanner using a 24-channel head array Nova coil (NOVA Medical Inc., Wilmington MA) and consisted of three sequences: a whole-brain MP2RAGE, a MP2RAGE covering a smaller slab^16,17^, and a multi-echo 3D FLASH^18^. The whole-brain MP2RAGE had 240 sagittal slices with an acquisition time of 10:57 min (repetition time (TR)=5,000 ms; echo time (TE)=2.45 ms; inversion times TI1/TI2=900/2,750 ms; flip angle=5°/3°; bandwidth=250 Hz/Px; voxel size=(0.7 mm)^3^; Table 2 (available online only)). The MP2RAGE slab consisted of 128 slices with an acquisition time of 9:07 min (TR=5,000 ms; TE=3.71 ms; TI1/TI2=900/2,750 ms; flip angle=5°/3°; bandwidth=240 Hz/Px; voxel size=(0.6 mm)^3^; Table 3 (available online only)). The FLASH slab consisted of 128 slices with an acquisition time of 17:18 min (TR=41 ms and three different echo times (TE): 11.22/20.39/29.57 ms; flip angle=14°; bandwidth=160 Hz/Px; voxel size=(0.5 mm)^3^; Table 4 (available online only)). Both slab sequences consisted of axial slices tilted −23 degrees to the true axial plane in scanner coordinates. This angle in combination with the used field of view ensured that the entire Basal Ganglia were scanned. To get a better inversion of the magnetization in the lower parts of the brain (e.g., the Cerebellum), a TR-FOCI inversion pulse was implemented in the MP2RAGE sequence^16^.

Unless indicated otherwise, all MRI data files were converted from DICOM to NIfTI format using an in-house dicom-to-nifti converter. This linux compatible converter is available via https://github.com/isis-group/isis.

### Scan volumes

The MP2RAGE sequence results in four different volumes for each subject: INV1, INV2, UNI and T1. The INV1 volume reflects the gradient echo sequence with an inversion time of 900 ms. The INV2 volume reflects the gradient echo sequence with an inversion time of 2,750 ms. The UNI volume is the combined volume of the two inversion times. Finally, the T1 volume is a T1 estimation map derived from the two inversion times (Marques *et al.*^17^). The FLASH sequence results in two different volumes per echo time per subject resulting in nine different volumes in total. Besides the standard T2* weighted magnitude image, the phase images are also provided and can be used to calculate susceptibility weighted images as well as quantitative susceptibility maps (e.g., Deistung *et al.*^19^).

### Data processing

**Figure 1:**
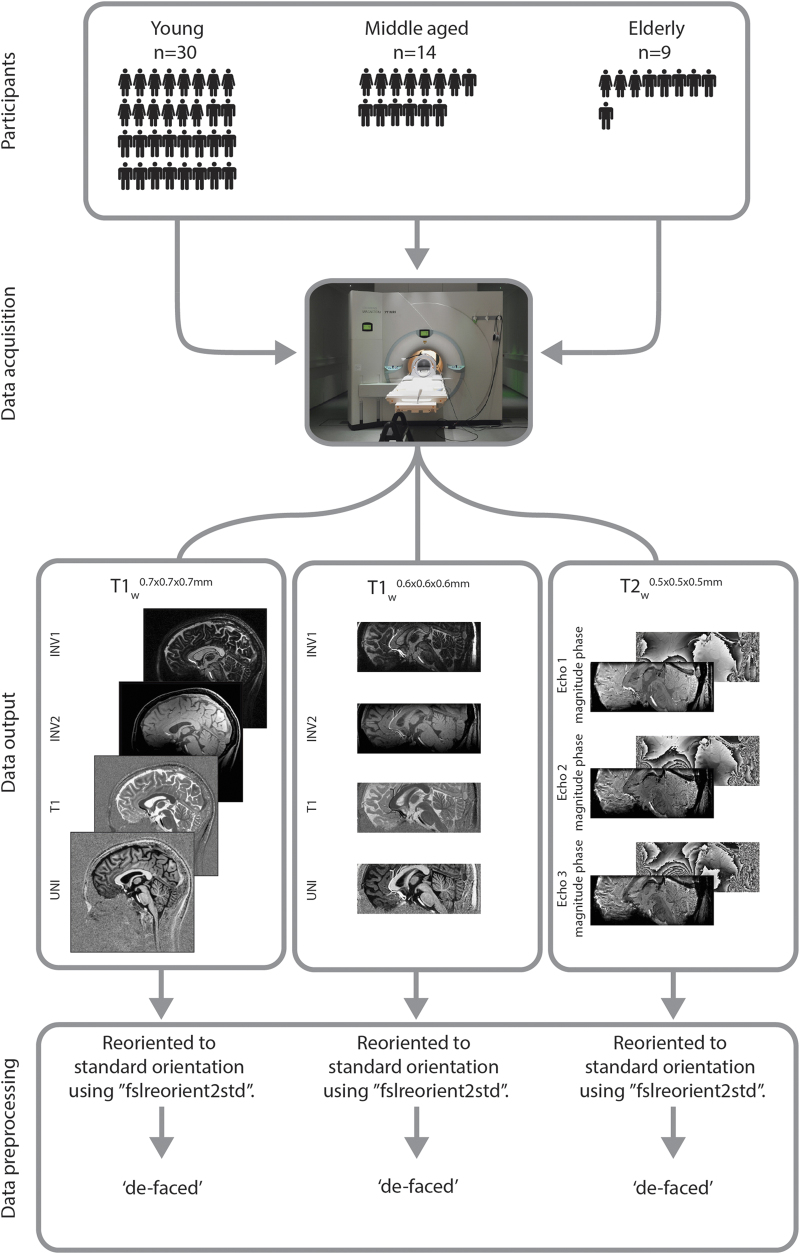
Data acquisition workflow. Three different age groups were structurally scanned using a 7 T MRI scanner. Data acquisition was done in a single imaging session that lasted for approximately 37 min. This resulted in three different datasets: a whole brain T1-weighted MP2RAGE volume; a slab T1-weighted MP2RAGE volume, and a T2*-weighted flash volume. All structural data was anonymized and reoriented to standard MNI orientation (7 T MRI photo courtesy of Andreas Döring).

All structural scans were anonymized by zeroing out the voxels in the vicinity of the facial surface, teeth, and auricles following a similar procedure as described by Hanke *et al.*^20^ All data were reoriented to the standard MNI space using the fslreorient2std tool as implemented in fslutils 5.0.2 (Figure 1).

## Data Records

All data records listed in this section are available from NITRIC (Data Citation 1) or Dryad (Data Citation 2). A README file with a detailed description of the content of all downloads is available in Dryad. Additional material and information are also provided in Data Citation 1 and Data Citation 2.

Unless noted otherwise, all MRI data files were converted from DICOM to NIfTI format using an in-house dicom-to-nifti converter. In order to de-identify data, information on centre-specific study and participant codes have been removed using an automated procedure. All human participants were given sequential integer IDs.

### Technical Validation

#### Motion artifacts

**Figure 2:**
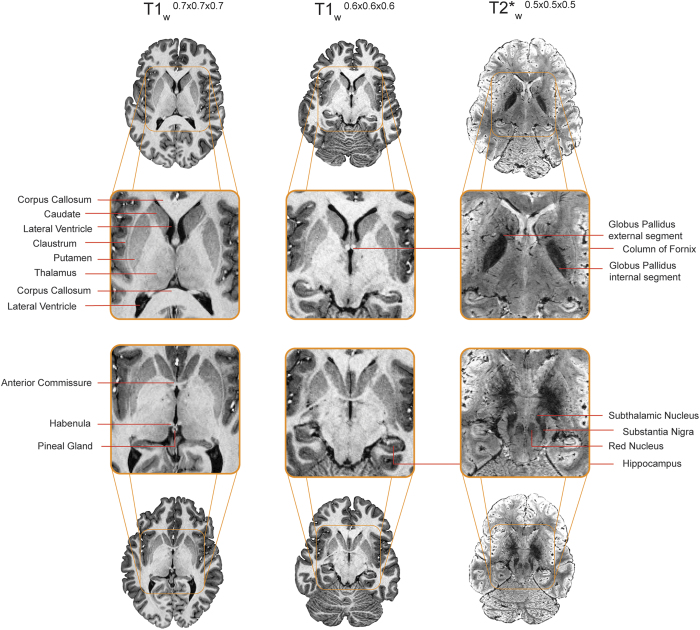
An example of the data quality. Two axial images of the three acquired datasets are displayed for a representative young subject. Only a few of the easily identifiable structures have been labeled. Note that not all structures are equally well visibly in the T1-weighted volumes compared to the T2*-weighted volume and argue for the need of multi sequence acquisition when interested in subcortical structures.

In line with Gedamu *et al.*^21^, motion artifacts in the structural volumes were estimated by calculating the noise ratio between the phase encoding direction and read direction outside of the brain. Two ROIs of +/−1,225 mm^2^ was drawn in the sagittal plane; 5 mm lateral of the skull, and in the coronal plane; 5 mm anterior of the skull, in the magnitude image of the second inversion time of the MP2RAGE sequence and FLASH sequences. The sagittal ROI corresponds to the read direction for the MP2RAGE whole brain and phase encoding direction for the MP2RAGE and FLASH slab, whereas the coronal ROI corresponds to the phase encoding direction for the MP2RAGE whole brain and read direction for the MP2RAGE and FLASH slab. The mean signal was extracted from both ROI’s and the mean phase encoding direction signal was divided by the mean read direction signal. The closer this ratio is to 1, the less motion artifacts are present. Following Gedamu *et al.*^21^, we estimated that any ratio below 2 reflects little to no motion artifacts (see Figure 2 for an example of the data quality).

One sided *t*-tests were conducted to test whether any of the groups showed significant motion artifacts in any of the sequences. All ratios per sequence and age group were significantly lower than 2 (MP2RAGE whole-brain: young (t(29)=−17.93, *P*<0.001); middle-aged (t(13)=−5.44, *P*<0.001); elderly (t(8)=−7.19, *P*<0.001), MP2RAGE slab: young (t(29)=−35.06, *P*<0.001); middle-aged (t(13)=−23.43, *P*<0.001); elderly (t(8)=−13.33, *P*<0.001), FLASH echo 1: young (t(29)=−3.74, *P*<0.001); middle-aged (t(13)=−17.68, *P*<0.001); elderly (t(8)=−16.97, *P*<0.001), FLASH echo 2: young (t(29)=−6.88, *P*<0.001); middle-aged (t(13)=−14.88, *P*<0.001); elderly (t(8)=−6.31, *P*<0.001), FLASH echo 3: young (t(29)=−10.36, *P*<0.001); middle-aged (t(13)=−6.23, *P*<0.001); elderly (t(8)=−19.53, *P*<0.001); Table 5 (available online only)).

There was no main effect of age on motion for the MP2RAGE whole brain (F(2,50)=1.29, *P*=0.29) or MP2RAGE slab (F(2,50)=0.8, *P*=0.44). There was a main effect of age and echo time on motion for the FLASH sequence (age: F(2,147)=4.97, *P*=0.008, echo time: F(2,147)=10.45, *P*<0.001). *Post-hoc* testing showed that the young had significantly more motion artifacts than both the middle-aged and elderly (young versus middle-aged: t(103.18)=5.61, *P*<0.001, young versus elderly: t(79.03)=5.25, *P*<0.001) whereas the middle-aged and elderly did not differ significantly (t(65.73)=−0.59, *P*=1.0). *Post-hoc* testing showed that the first echo time had significantly less motion artifacts than both the second and third echo time (first echo versus second echo: t(90)=−3.29, *P*=0.003, first echo versus third echo: t(82.17)=−3.77, *P*=0.001) whereas the second and third echo time did not differ significantly (t(101.66)=−0.72, *P*=0.92). All *post-hoc* testing was Bonferroni corrected at an alpha of 0.05.

### Signal to noise ratio

To estimate the Signal to Noise Ratio (SNR), the mean signal from an axial slice just above the corpus callosum was divided by the standard deviation of the signal in the read direction ROI both in the magnitude image of the second inversion time of the MP2RAGE sequence and FLASH sequences. To improve the estimation of noise a Rician correction was applied^22^. As this is still an approximation of the true SNR, the term SNR_approx_. is used. For the three different sequences there was a main effect of age on SNR_approx_. (MP2RAGE whole brain: F(2,50)=48.3, *P*<0.001; MP2RAGE slab: F(2,50)=5.94, *P*=0.005; FLASH: F(2,147)=6.90, *P*=0.001). Additionally there was a main effect of echo time on SNR_approx_. (F(2,147)=11.75, *P*<0.001).

*Post-hoc* testing showed that for the MP2RAGE whole brain, the young had a significantly higher SNR_approx_ than both the middle-aged and elderly (young versus middle-aged: t(33.72)=8.87, *P*<0.001; young versus elderly: t(18.37)=8.41, *P*<0.001) whereas the middle-aged and elderly did not differ significantly (t(17.61)=0.46, *P*=1.0). A similar pattern was found for the MP2RAGE slab. The young had a significantly higher SNR_approx_ than both the middle-aged and elderly (young versus middle-aged: t(24.8)=2.86, *P*=0.017; young versus elderly: t(17.46)=2.92, *P*=0.019) whereas the middle-aged and elderly did not differ significantly (t(20.56)=−0.10, *P*=1.0). The young had a significantly higher SNR_approx_ in the FLASH sequence than the middle-aged (t(70.80)=3.35, *P*=0.003) but did not differ from the elderly (t(36.31)=0.16, *P*=1.0). The middle-aged and elderly did not differ in SNR_approx_ for the FLASH sequence (t(51.87)=−2.16, *P*=0.071). *Post-hoc* testing showed that the first echo time had significantly more SNR_approx_ than both the second and third echo time (first echo versus second echo: t(97.2)=4.89, *P*<0.001, first echo versus third echo: t(88.4)=8.05, *P*<0.001). The second echo time had significantly higher SNR_approx_ than the third echo time (t(100.91)=3.42, *P*=0.002). All *post-hoc* testing was Bonferroni corrected at an alpha of 0.05 (Table 6 (available online only)).

In addition to the SNR_approx_. calculation and the noise ratio between the phase encoding direction and read direction, the scans were visually inspected by two independent researchers. The FLASH magnitude scans were checked for ghosting, wrapping, or shading artifacts. The MP2RAGE UNI scans were checked for ghosting, wrapping, shading, and the presence of ‘zebra stripe’ artifacts. Finally the MP2RAGE T1 scans were checked for ghosting, wrapping, shading, the presence of ‘zebra stripes’, and CSF clipping artifacts where ‘1’ corresponds to not present at all and ‘5’ corresponds to severely present.

Ghosting artifacts are generally caused by motion and appear as a ‘ghost’ image of the brain in phase encoding direction. Wrapping artifacts are usually caused by anatomical features protruding outside of the imaged field of view but still within the sensitive volume of the RF coil. Shading artifacts were defined as a non-homogenous intensity throughout the entire brain. Zebra stripes were defined as well defined alternating black and white stripes present in the brain. Finally, CSF clipping artifacts were defined as the voxels in the CSF that have a signal dropout and appear black (McRobbie *et al.,* 2006).

The mean rating for each scale for each checked volume is given in Table 7 (available online only). Volumes that had a higher rating on that quality check than the rest of the age group based on the +/−1.5* interquartile range are highlighted with an asterisk.

As a result of the scan parameters of the MP2RAGE sequence, a number of participants show T1 clipping artifacts in the T1 map located in the CSF. This is indicated in Table 7 (available online only). Note that these clipping artifacts do not affect the T1 values reported in the grey and white matter tissue.

## Usage Notes

The procedures we employed in this study resulted in a dataset that is highly suitable for automated processing. Data are shared in documented standard formats, such as NIfTI or plain text files, to enable further processing in arbitrary analysis environments with no imposed dependencies on proprietary tools. All processing performed on the released data article were produced by open-source software on standard computer workstations.

## Additional information

Tables 2, 3, 4, 5, 6, 7 are only available in the online version of this paper.

**How to cite this article:** Forstmann, B. U. *et al.* Multi-modal ultra-high resolution structural 7-Tesla MRI data repository. *Sci. Data* 1:140050 doi: 10.1038/sdata.2014.50 (2014).

## Supplementary Material



## Figures and Tables

**Table 1:** Demographic information of participants.

Ag**e Group**	**Participant**	**Gender**	**Age**
1	pp01	Female	23
	pp02	Female	23
	pp03	Female	25
	pp04	Female	23
	pp05	Male	27
	pp06	Female	23
	pp07	Male	27
	pp08	Female	24
	pp09	Male	24
	pp10	Male	22
	pp11	Female	25
	pp12	Female	24
	pp13	Male	24
	pp14	Male	26
	pp15	Male	23
	pp16	Female	25
	pp17	Female	19
	pp18	Male	23
	pp19	Male	21
	pp20	Male	25
	pp21	Male	24
	pp22	Male	28
	pp23	Male	28
	pp24	Female	22
	pp25	Female	19
	pp26	Female	21
	pp27	Male	25
	pp28	Female	21
	pp29	Male	26
	pp30	Male	23
2	pp31	Female	56
	pp32	Female	60
	pp33	Female	58
	pp34	Male	40
	pp35	Male	42
	pp36	Male	60
	pp37	Female	59
	pp38	Female	49
	pp39	Female	45
	pp40	Female	55
	pp41	Male	55
	pp42	Male	49
	pp43	Male	54
	pp44	Male	53
3	pp45	Female	74
	pp46	Male	63
	pp47	Female	62
	pp48	Male	72
	pp49	Male	67
	pp50	Male	75
	pp51	Male	69
	pp52	Male	68
	pp53	Female	73

**Table 2:** Exam card MP2RAGE whole-brain scan

General	
TA	10:57
PAT	2
Voxel size	0.7×0.7×0.7 mm
Rel. SNR	1.0
	
**Properties**	
Prio Recon	Off
Load to viewer	On
Inline movie	Off
Auto store images	On
Load to stamp segments	Off
Load images to graphic segments	Off
Auto open inline display	Off
Start measurement without further preparation	On
Wait for user to start	Off
Start measurements	single
	
**Routine**	
Slab group 1	
Slabs	1
Dist. factor	50%
Position	Isocenter
Orientation	Sagittal
Phase enc. dir.	A >> P
Rotation	0.00 deg
Phase oversampling	0%
Slice oversampling	0.0%
Slices per slab	240
FoV read	224 mm
FoV phase	100.0%
Slice thickness	0.70 mm
TR	5,000 ms
TE	2.45 ms
Averages	1
Concatenations	1
Filter	None
Coil elements	A24
	
**Contrast**	
Magn. Preparation	Non-sel. IR
TI 1	900 ms
TI 2	2,750 ms
Flip angle 1	5 deg
Flip angle 2	3 deg
Fat suppr.	Water excit. normal
Water suppr.	None
2nd Inversion-Contrast	On
Averaging mode	Long term
Reconstruction	Magnitiude
Measurements	1
Multiple series	Each measurement
	
**Resolution**	
Base resolution	320
Phase resolution	100%
Slice resolution	100%
Phase partial Fourier	6/8
Slice partial Fourier	Off
Interpolation	Off
PAT mode	GRAPPA
Accel. factor PE	2
Ref. lines PE	24
Accel. factor 3D	1
Reference scan mode	Integrated
Image Filter	Off
Distortion Corr.	Off
Prescan Normalize	Off
Normalize	Off
B1 filter	Off
Raw filter	Off
Elliptical filter	Off
	
**Geometry**	
Multi-slice mode	Single shot
Series	Interleaved
Table position	H
Table position	0 mm
Inline Composing	Off
	
**System**	
V24	Off
A24	On
Positioning mode	FIX
MSMA	S-C-T
Sagittal	R >>L
Coronal	A>>P
Transversal	F>>H
Save uncombined	Off
Coil Combine Mode	Adaptive Combine
AutoAlign	---
Auto Coil Select	Off
Shim mode	Standard
Adjust with body coil	Off
Confirm freq. adjustment	Off
Assume Silicone	Off
? Ref. amplitude 1H	0.000 V
Adjustment Tolerance	Auto
Adjust volume	
Position	Isocenter
Orientation	Sagittal
Rotation	0.00 deg
F>>H	224 mm
A>>P	224 mm
R>>L	168 mm
	
**Physio**	
1st Signal/Mode	None
Dark blood	Off
Resp. control	Off
	
**Inline**	
Subtract	Off
Std-Dev-Sag	Off
Std-Dev-Cor	Off
Std-Dev-Tra	Off
Std-Dev-Time	Off
MIP-Sag	Off
MIP-Cor	Off
MIP-Tra	Off
MIP-Time	Off
Save original images	On
	
**Sequence**	
Introduction	On
Dimension	3D
Elliptical scanning	Off
Asymmetric echo	Allowed
Contrasts	1
Bandwidth	250 Hz/Px
Flow comp.	No
Echo spacing	6.8 ms
RF pulse type	Fast
Gradinet mode	Fast
Excitation	Non-sel.
RF spoiling	On
FFT Scale Factor	100%
Line/Partition Swap	Off
Homodyne Phase Filter	Off
Flat Image	On
T1 Map	On
Division Image	Off
ExtInvPulseOn	On
OffResFreqInv	0
Invflipangle	1,700

**Table 3:** Exam card MP2RAGE slab.

General	
TA	9:07
PAT	2
Voxel size	0.6×0.6×0.6 mm
Rel. SNR	1.0
	
**Properties**	
Prio Recon	Off
Load to viewer	On
Inline movie	Off
Auto store images	On
Load to stamp segments	Off
Load images to graphic segments	Off
Auto open inline display	Off
Start measurement without further preparation	On
Wait for user to start	Off
Start measurements	single
	
**Routine**	
Slab group 1	
Slabs	1
Dist. factor	50%
Position	R2.4 A29.1 H23.0
Orientation	T>C-23.0
Phase enc. dir.	R>>L
Rotation	90.00 deg
Phase oversampling	0%
Slice oversampling	100.0%
Slices per slab	128
FoV read	192 mm
FoV phase	81.3%
Slice thickness	0.60 mm
TR	5,000 ms
TE	3.71 ms
Averages	1
Concatenations	1
Filter	None
Coil elements	A24
	
**Contrast**	
Magn. Preparation	Non-sel. IR
TI 1	900 ms
TI 2	2,750 ms
Flip angle 1	5 deg
Flip angle 2	3 deg
Fat suppr.	None
Water suppr.	None
2nd Inversion-Contrast	On
Averaging mode	Long term
Reconstruction	Magnitiude
Measurements	1
Multiple series	Each measurement
	
**Resolution**	
Base resolution	320
Phase resolution	100%
Slice resolution	100%
Phase partial Fourier	6/8
Slice partial Fourier	6/8
Interpolation	Off
PAT mode	GRAPPA
Accel. factor PE	2
Ref. lines PE	24
Accel. factor 3D	1
Reference scan mode	Integrated
Image Filter	Off
Distortion Corr.	Off
Prescan Normalize	Off
Normalize	Off
B1 filter	Off
Raw filter	Off
Elliptical filter	Off
	
**Geometry**	
Multi-slice mode	Single shot
Series	Interleaved
Table position	H
Table position	0 mm
Inline Composing	Off
	
**System**	
V24	Off
A24	On
Positioning mode	FIX
MSMA	S-C-T
Sagittal	R >>L
Coronal	A>>P
Transversal	F>>H
Save uncombined	Off
Coil Combine Mode	Adaptive Combine
AutoAlign	---
Auto Coil Select	Off
Shim mode	Standard
Adjust with body coil	Off
Confirm freq. adjustment	Off
Assume Silicone	Off
Ref. amplitude 1H	0.000 V
Adjustment Tolerance	Auto
Adjust volume	
Position	R2.4 A29.1 H23.0
Orientation	T>C-23.0
Rotation	90.00 deg
A>>P	192 mm
R>>L	156 mm
F>>H	77 mm
	
**Physio**	
1st Signal/Mode	None
Dark blood	Off
Resp. control	Off
	
**Inline**	
Subtract	Off
Std-Dev-Sag	Off
Std-Dev-Cor	Off
Std-Dev-Tra	Off
Std-Dev-Time	Off
MIP-Sag	Off
MIP-Cor	Off
MIP-Tra	Off
MIP-Time	Off
Save original images	On
	
**Sequence**	
Introduction	On
Dimension	3D
Elliptical scanning	Off
Asymmetric echo	Allowed
Contrasts	1
Bandwidth	240 Hz/Px
Flow comp.	Slice
Echo spacing	7.5 ms
RF pulse type	Fast
Gradinet mode	Whisper
Excitation	Slab-sel.
RF spoiling	On
FFT Scale Factor	100%
Line/Partition Swap	Off
Homodyne Phase Filter	Off
Flat Image	On
T1 Map	On
Division Image	Off
ExtInvPulseOn	On
OffResFreqInv	0
Invflipangle	1,800

**Table 4:** Exam card flash slab

General	
TA	17:18
PAT	Off
Voxel size	0.5×0.5×0.5 mm
Rel. SNR	1.0
	
**Properties**	
Prio Recon	Off
Load to viewer	On
Inline movie	Off
Auto store images	On
Load to stamp segments	Off
Load images to graphic segments	Off
Auto open inline display	Off
Start measurement without further preparation	On
Wait for user to start	Off
Start measurements	single
	
**Routine**	
Slab group 1	
Slabs	1
Dist. factor	20%
Position	R2.4 A29.1 H23.0
Orientation	T>C-23.0
Phase enc. dir.	R>>L
Rotation	90.00 deg
Phase oversampling	0%
Slice oversampling	12.5%
Slices per slab	128
FoV read	192 mm
FoV phase	81.3%
Slice thickness	0.50 mm
TR	41 ms
TE 1	11.22 ms
TE 2	20.39 ms
TE 3	29.57 ms
Averages	1
Concatenations	1
Filter	None
Coil elements	A24
	
**Contrast**	
MTC	Off
Magn. preperation	None
Flip angle	14 deg
Fat suppr.	None
Water suppr.	None
SWI	Off
Averaging mode	Short term
Reconstruction	Magn./Phase
Measurements	1
Multiple series	Each measurement
	
**Resolution**	
Base resolution	384
Phase resolution	100%
Slice resolution	100%
Phase partial Fourier	6/8
Slice partial Fourier	6/8
Interpolation	Off
PAT mode	None
Image Filter	Off
Distortion Corr.	Off
Prescan Normalize	Off
Normalize	Off
B1 filter	Off
Raw filter	Off
Elliptical filter	Off
	
**Geometry**	
Multi-slice mode	Interleaved
Series	Interleaved
Saturation mode	Standard
Special sat.	None
Table position	H
Table position	0 mm
Inline Composing	Off
Tim CT mode	Off
	
**System**	
V24	Off
A24	On
Positioning mode	REF
MSMA	S-C-T
Sagittal	R >>L
Coronal	A>>P
Transversal	F>>H
Save uncombined	Off
Coil Combine Mode	Adaptive Combine
AutoAlign	---
Auto Coil Select	Off
Shim mode	Standard
Adjust with body coil	Off
Confirm freq. adjustment	Off
Assume Silicone	Off
? Ref. amplitude 1H	0.000 V
Adjustment Tolerance	Auto
Adjust volume	
Position	R2.4 A29.1 H23.0
Orientation	T>C-23.0
Rotation	90.00 deg
A>>P	192 mm
R>>L	156 mm
F>>H	64 mm
	
**Physio**	
1st Signal/Mode	None
Segments	1
Tagging	None
Dark blood	Off
Resp. control	Off
	
**Inline**	
Subtract	Off
Liver registration	Off
Std-Dev-Sag	Off
Std-Dev-Cor	Off
Std-Dev-Tra	Off
Std-Dev-Time	Off
MIP-Sag	Off
MIP-Cor	Off
MIP-Tra	Off
MIP-Time	Off
Save original images	On
Wash – In	Off
Wash – Out	Off
TTP	Off
PEI	Off
MIP – time	Off
MapIt	None
Contrasts	3
	
**Sequence**	
Introduction	On
Dimension	3D
Elliptical scanning	Off
Phase stabilization	On
Asymmetric echo	Off
Bandwidth 1	160 Hz/Px
Bandwidth 2	160 Hz/Px
Bandwidth 3	160 Hz/Px
Flow comp. 1	Yes
Flow comp. 2	No
Flow comp. 3	No
Readout mode	Monopolar
RF pulse type	Normal
Gradieet mode	Whisper
Excitation	Slab-sel.
RF spoiling	On
length exc pulse	3,000 us
Ernst Angle?	On
T1	1,300 ms
FFT scale factor	2.5

**Table 5:** Noise ratio between the phase encoding direction and read direction per participant

**Age Group**	**Participant ID**	**MP2RAGE whole brain**	**MP2RAGE slab**	**FLASH echo 1**	**FLASH echo 2**	**FLASH echo 3**
1	pp01	1.79	1.22	1.45	1.84	2.02
	pp02	1.25	0.85	1.27	1.56	1.50
	pp03	1.34	0.95	0.93	1.10	1.12
	pp04	1.46	1.13	1.19	1.80	2.05
	pp05	2.33	0.65	1.31	1.92	2.13
	pp06	1.10	1.07	1.84	2.29	2.57
	pp07	1.60	1.06	1.59	2.46	2.98*
	pp08	1.83	0.97	1.11	1.42	1.48
	pp09	1.52	0.85	0.51	0.75	1.03
	pp10	1.83	1.13	1.48	1.95	1.90
	pp11	1.45	0.95	1.05	1.47	1.52
	pp12	1.51	1.10	1.46	1.77	1.77
	pp13	1.34	1.01	0.97	1.29	1.41
	pp14	1.84	0.81	0.94	1.41	1.55
	pp15	1.61	0.96	0.98	1.29	1.41
	pp16	1.19	1.11	1.30	1.58	1.52
	pp17	1.56	1.26	1.68	2.42	2.61
	pp18	1.69	0.67	0.59	0.80	0.96
	pp19	1.00	0.82	1.60	1.81	1.84
	pp20	1.33	0.93	1.03	1.60	1.82
	pp21	1.99	0.98	0.80	1.18	1.71
	pp22	1.25	0.83	1.28	1.48	1.45
	pp23	1.78	0.87	0.97	1.40	1.60
	pp24	1.36	1.03	0.97	1.34	1.29
	pp25	1.46	1.12	1.26	1.99	2.29
	pp26	1.84	0.98	1.27	1.50	1.35
	pp27	1.44	0.74	0.92	1.01	0.97
	pp28	1.36	1.25	0.85	1.35	1.41
	pp29	1.35	0.77	0.53	0.46*	0.45*
	pp30	1.58	0.93	0.63	0.63	0.71
2	pp31	1.46	1.01	0.82	1.37	1.80
	pp32	1.21	1.22*	1.36*	1.90	2.01
	pp33	1.11	0.99	0.68	0.61	0.56
	pp34	0.98	1.01	0.88	1.20	1.35
	pp35	1.91	1.07	1.36*	1.12	0.90
	pp36	1.75	1.30*	0.59	0.60	0.59
	pp37	1.36	1.04	0.84	1.01	1.10
	pp38	1.22	1.03	0.87	1.22	1.32
	pp39	2.23	1.09	0.66	0.62	0.65
	pp40	1.34	1.00	0.71	0.68	0.60
	pp41	1.18	0.60*	0.58	0.57	0.61
	pp42	1.77	0.88	0.68	0.78	0.73
	pp43	1.68	0.98	1.18	1.42	1.43
	pp44	1.61	0.97	0.69	0.83	0.81
3	pp45	1.10	0.96	1.10	1.13	1.16
	pp46	1.81	0.81	0.81	1.09	1.15
	pp47	1.62	1.39	0.99	1.10	0.98
	pp48	1.16	1.35	1.23	1.74*	1.88*
	pp49	1.19	0.86	1.00	0.84	0.70
	pp50	1.53	1.04	0.69	0.81	0.90
	pp51	1.40	1.16	1.17	1.06	0.88
	pp52	1.36	0.84	0.60	0.79	0.89
	pp53	0.97	1.00	0.81	0.84	0.84

*indicates participants displaying more noise than the rest of the age group based on the +/−1.5* interquartile range.

**Table 6:** SNR_approx_ between the axial slab containing the brain and read direction per participant

**Age Group**	**Participant ID**	**MP2RAGE whole brain**	**MP2RAGE slab**	**FLASH echo 1**	**FLASH echo 2**	**FLASH echo 3**
1	pp01	74.07	36.26	28.31	19.74	13.88
	pp02	68.04	32.93	31.55	25.20	21.28
	pp03	71.61	32.65	43.90	34.04	28.94
	pp04	68.41	38.57	25.47	17.84	13.45
	pp05	68.70	19.62	21.72	14.63	10.79
	pp06	57.61	37.66	24.11	16.20	11.69
	pp07	69.46	30.72	14.33	10.30	7.67
	pp08	72.91	36.63	33.06	25.17	20.39
	pp09	52.87	21.00	37.48	27.86	21.44
	pp10	67.00	31.57	24.50	18.15	14.61
	pp11	70.31	37.40	32.28	25.54	21.60
	pp12	71.88	42.04	28.77	20.01	15.65
	pp13	47.83	33.44	32.85	22.23	18.73
	pp14	63.25	26.36	25.58	20.72	17.19
	pp15	63.64	28.77	31.36	20.07	15.19
	pp16	67.87	44.97	35.20	25.34	19.96
	pp17	68.88	37.52	20.63	15.21	11.89
	pp18	45.70	15.66	36.40	25.75	20.38
	pp19	49.77	28.37	28.29	18.96	14.16
	pp20	52.66	27.79	22.42	18.43	15.62
	pp21	61.11	21.16	18.67	14.59	11.71
	pp22	67.61	26.28	31.54	23.89	20.69
	pp23	57.75	25.50	29.21	20.35	15.52
	pp24	72.25	37.29	34.14	28.39	25.00
	pp25	61.48	30.45	24.30	16.17	12.18
	pp26	68.43	42.36	33.85	26.21	23.52
	pp27	59.68	22.90	27.11	19.03	14.64
	pp28	72.48	40.40	33.42	26.92	22.39
	pp29	40.59	24.11	29.65	19.52	15.88
	pp30	70.26	34.31	32.35	24.13	17.07
2	pp31	42.74	31.62	18.92	14.60	12.21
	pp32	51.47	35.65	23.58	17.31	13.67
	pp33	37.90	24.76	31.52	23.11	20.73
	pp34	32.31	23.43	34.78	24.29	18.90
	pp35	53.76	12.87	4.41	5.77	6.55
	pp36	43.26	20.98	13.67	10.37	7.87
	pp37	44.84	30.65	41.02	28.54	21.25
	pp38	46.50	28.15	33.05	24.69	19.84
	pp39	29.26	29.54	19.72	14.14	11.75
	pp40	42.61	33.55	28.84	21.36	18.10
	pp41	40.77	11.39	15.05	10.16	6.85
	pp42	42.05	15.26	14.55	11.70	10.59
	pp43	42.97	19.59	13.93	10.57	8.30
	pp44	34.88	25.06	21.02	14.52	11.16
3	pp45	33.50	30.38	41.46	34.87	27.63
	pp46	35.78	21.28	33.06	21.70	17.80
	pp47	44.54	31.90	13.45	9.51	7.46
	pp48	35.19	24.80	20.81	14.74	11.96
	pp49	37.68	29.37	25.39	19.94	17.02
	pp50	39.52	24.08	32.32	23.55	18.50
	pp51	47.61	16.97	15.18	12.67	10.58
	pp52	52.99	16.65	37.19	30.15	23.64
	pp53	37.86	27.35	35.00	24.54	19.52

**Table 7:** The mean artifact rating between two raters

**Age group**	**Flash**				**MP2RAGE Slab**					**MP2RAGE Brain**				
	**Subjects**	**Ghosting**	**Wrapping**	**Shading**	**Ghosting**	**Wrapping**	**Shading**	**Zebra stripes**	**Clipping**	**Ghosting**	**Wrapping**	**Shading**	**Zebra stripes**	**Clipping**
*`1*	*pp01*	1	1	1,5	1	1,5	3	1	1	1	1	3	1	1
	*pp02*	1	1	4	1	1,5	3	1	1	1	1	3	1	1
	*pp03*	1	1	2	1	1,5	2,5	1	1	1	1	3	1	3,5
	*pp04*	1	1	3,5	1	1,5	2,5	1	1	1	1	3,5	1	4
	*pp05*	1	1	2	1	1,5	2,5	1	1	1	1	3	1	2,5
	*pp06*	1	1	3,5	1	1,5	4	1	1	1	1,5	3	1	4,5
	*pp07*	1	1	3,5	1	1,5	4	1	1	1	1	3	1	3
	*pp08*	1	1	2	1	1,5	4	1	1	1	1	3,5	1	2,5
	*pp09*	1	1	4,5	1	1,5	4,5	1	1	1	1	3,5	1	3
	*pp10*	1	2	2,5	1	1,5	4,5	1	1	1	1	4	1	4
	*pp11*	1	1,5	2	1	1,5	3,5	1	1	1	1	3	1	4
	*pp12*	1	1	2	1	1,5	4	1	1	1,5*	1	3	1	2,5
	*pp13*	1	1	2,5	1	1,5	2,5	1	1	1	1	3	1	2,5
	*pp14*	1,5*	1	4	1	1,5	4	1	1	1	1	3	1	2,5
	*pp15*	1	1,5	1,5	1	1,5	3,5	1	1	1,5*	1	3	1	2,5
	*pp16*	1	1	2	1	1,5	3,5	1	1	1,5*	1	3	1	2,5
	*pp17*	1	1	3,5	1	1,5	3,5	1	1	1	1	3	3*	4
	*pp18*	1	4*	2,5	1	1,5	4,5	1	3	1	1	3	1	2,5
	*pp19*	1	1	3,5	1	1,5	4	1	2	1	1	3,5	1	1
	*pp20*	1,5*	1,5	4,5	1	1,5	4	1	2	1	1,5	3,5	2,5*	1,5
	*pp21*	1	1	4,5	1	1,5	4	1	1	1	1	3,5	1	1
	*pp22*	1	1	2	1	1,5	3,5	1	1	1	1	3	1	4
	*pp23*	1	2	4	1	1,5	4	1	2	1	1,5	3	1	1
	*pp24*	1	1	3,5	1	1,5	3,5	1	2	1,5*	1	3,5	1	1
	*pp25*	1	2	4	1	1,5	3,5	1	2	1	1,5	3	1	1
	*pp26*	1	1	4	1	1,5	4	1	2	1	1	3	1	4
	*pp27*	1	1	4	1	1,5	3,5	1	2	1	1	3	1	1
	*pp28*	1,5*	2	3,5	1	1,5	3,5	1	2	1,5*	1,5	3	3,5*	1
	*pp29*	1	2	4	1	1,5	3,5	1	2	1	1,5	3	1	4
	*pp30*	1	1	4	1	1,5	3,5	1	2	1	3*	3	1	2
*2*	*pp31*	1,5*	1	3,5	1	1,5	3,5	1	1	1	1,5	3	1	1
	*pp32*	1	1	3,5	1	1,5	3,5	1	1	1	1,5	3,5	2,5*	4,5
	*pp33*	1	1	3,5	1	1,5	3,5	1	2,5	1	1	3	1	3
	*pp34*	1	3,5*	4	1	2*	3,5	1	2,5	1,5*	1	3,5	1	4
	*pp35*	2,5*	3*	3,5	1	1,5	3,5	1	2,5	1,5*	1	3	1	4
	*pp36*	2,5*	2,5*	3,5	1	1,5	3,5	1	2,5	1	1	3,5	1	1
	*pp37*	2,5*	2,5*	3,5	1	1,5	3,5	1	1	1	1,5	3	1	1
	*pp38*	1	2,5*	3,5	1	1,5	3,5	1	1	1	1	3,5	1	1
	*pp39*	1	1	3,5	1	1,5	3,5	1	1	1	1	3,5	1	1
	*pp40*	1	1	3,5	1	1,5	3,5	1	1	1	1	3,5	1	1
	*pp41*	1,5*	2,5*	4	1	1,5	3	1	1	1	1	3,5	1	1
	*pp42*	2,5*	1	3,5	1	1,5	3,5	1	1	1	1,5	3,5	1	1
	*pp43*	1	1	4	1	3,5*	3,5	1	2,5	1	1,5	3	1	3
	*pp44*	1	1	4,5	1	1,5	3,5	1	1	1	1,5	3,5	1	2
*3*	*pp45*	1	1,5	4	1	1,5	3,5	1	1	1	1,5	3	1	4,5
	*pp46*	1	1,5	4	1	1,5	4	1	2,5	1	1,5	3,5	1	1
	*pp47*	1	1	4	1	1,5	3,5	1	1	1	1,5	3	1	1
	*pp48*	1	1,5	4	1	1,5	4	1	1	1	1,5	4	1	1
	*pp49*	1	1,5	4	1	1,5	3,5	1	2,5	1	2	3,5	1	1
	*pp50*	1	1	4,5	1	1,5	3	1	1	1	1	4	1	1
	*pp51*	1	1,5	4	1	1,5	4	1	2,5	1	1	3,5	1	1
	*pp52*	1,5*	1,5	4,5	1	1,5	3,5	1	1	1	1,5	3,5	1	4,5
	*pp53*	1	1	4	1	1,5	3,5	1	2,5	1,5*	1	3	1	1
